# *Gundelia tournefortii* Antidiabetic Efficacy: Chemical Composition and GLUT4 Translocation

**DOI:** 10.1155/2018/8294320

**Published:** 2018-04-26

**Authors:** Sleman Kadan, Yoel Sasson, Bashar Saad, Hilal Zaid

**Affiliations:** ^1^Qasemi Research Center, Al-Qasemi Academic College, P.O. Box 124, 30100 Baqa Al-Gharbiyye, Israel; ^2^Casali Center for Applied Chemistry, Institute of Chemistry, The Hebrew University of Jerusalem, Givat Ram, 91904 Jerusalem, Israel; ^3^Faculty of Sciences, Arab American University, P.O. Box 240, Jenin, State of Palestine

## Abstract

In the present* in vitro* study, we tested the chemical composition, cytotoxicity and antidiabetic activity of two distinct extracts of wild Artichoke-like vegetable,* Gundelia tournefortii*: methanol and hexane. GC/MS phytochemical analysis of* G. tournefortii *methanol and hexane extracts revealed 39 compounds reported here for the first time in* G. tournefortii *out of the 45 detected compounds. Only Stigmasterol was present in both extracts. The efficacy of* G. tournefortii *extracts in enhancing glucose transporter 4 (GLUT4) translocation to the plasma membrane (PM) was tested in L6 muscle cells stably expressing myc-tagged GLUT4 (L6-GLUT4myc) using cell-ELISA test. Results obtained here indicate that methanol and hexane extracts were safe up to 250 *μ*g/ml as measured with MTT and the LDH leakage assays. The methanol extract was the most efficient in GLUT4 translocation enhancement. It increased GLUT4 translocation at 63 *μ*g/ml 1.5- and 2-fold relative to the control in the absence and presence of insulin, respectively. These findings indicate that* G. tournefortii *possesses antidiabetic activity in part by enhancing GLUT4 translocation to the PM in skeletal muscle.

## 1. Introduction

Plants produce a remarkably diverse array of thousands of secondary metabolites. In addition to their roles in the defense of plants against changing environmental conditions, they were reported to be beneficial in treating animals and human being diseases [1–4]. The phytochemicals, polysaccharides, flavonoids [5], terpenoids, tannins and steroids [6], and others, were reported to possess antidiabetic activity [2]. Metformin and resveratrol, two main antidiabetic drugs, were derived from medicinal plants [7].

Circulating glucose levels can be balanced through controlling glucose production and utilization or through increasing insulin secretion and effectiveness as well as through increasing energy expenditure or reduction of energy intake [8, 9]. The natural herbs for diabetes treatment focus on lowering blood sugar and minimizing the damaging effects of the disease. The action mechanism(s) of antidiabetic plants are usually insulin mimics, sensitizer, and secretagogues as well as inhibitors of intestinal carbohydrate digestion and absorption [2, 3, 10, 11].

Insulin sensitizers include plants that increase glucose uptake and disposal by muscle, fat, and hepatic cells and those that regulate hepatic glycogen metabolism. For instance, garlic* (Allium sativum)* and onion* (Allium cepa)* were reported to decrease blood glucose levels by normalizing liver glucose-6-phosphatase and hexokinase activity [12]. Black cumin* (Nigella sativa) *and cinnamon* (Cinnamomum officinalis)* were suggested to have insulin mimetic properties, through enhancing insulin signaling pathway independently of insulin [13, 14]. We had recently tested several medicinal plants extracts mechanisms in increasing glucose uptake and found that* Trigonella foenum-graecum*,* Urtica dioica*,* Atriplex halimus*,* Cinnamomum officianalis*, and* Ocimum basilicum* increase glucose disposal by enhancing the glucose transporter 4 (GLUT4) translocation to the plasma membrane [9, 10].

Glucose uptake into skeletal muscle is mediated by the facilitative glucose transporter-4 (GLUT4), a membrane protein that continuously recycles between intracellular vessels and the plasma membrane (PM). Insulin primarily enhances the rate of GLUT4 exocytosis towards and fusion with the PM, a process termed GLUT4 translocation that results in a gain in PM GLUT4 [8].

Wild Artichoke-like vegetable,* Gundelia tournefortii*, is one of the traditional used antidiabetic herbs.* G. tournefortii *is an edible spiny, thistle-like plant native to the Middle East and other areas of Western Asia [15]. It is considered as one of the highest cultural importance valuable eatable wild species in Palestine [16]. In the Greco-Arab medicine,* G. tournefortii *has been used for different pathological conditions including inflammation and antibiotic resistant inhibitor [17], hepatoprotective and blood purifier [18], and hypolipidemic and antioxidant agent [15], as well as antidiabetes herb [19, 20].* G. tournefortii *antidiabetic activity was evaluated in dexamethasone induced diabetic mice. Oral administration of* G. tournefortii *to the dexamethasone induced diabetic mice led to a significant decrease in serum glucose levels (as well as triglyceride and cholesterol) [21].

The aim of the present study was to evaluate the chemical composition of* G. tournefortii *extracts by GC/MS and determine if GLUT4 translocation plays a role in its antidiabetic effect.

## 2. Materials and Methods

### 2.1. Materials

All tissue culture reagents including fetal bovine serum and standard culture medium *α*-MEM (modified Eagle's medium) were purchased from Biological Industries (Beit Haemek, Israel). Horseradish peroxidase (HRP)-conjugated goat anti-rabbit antibodies were obtained from Promega (Madison, WI, USA). Polyclonal anti-myc (A-14) and other standard chemicals were purchased from Sigma.

### 2.2. Plant Extract Preparation

*Gundelia tournefortii *(aerial parts) were collected from the Galil area in Israel in March 2016. Forty grams of the air-dried aerial parts of* G. tournefortii *were powdered and packed in an Erlenmeyer. The Erlenmeyers were then sonicated for 2 hours at 50°C and left in dark glass bottles for 24 h for complete extraction hours to give a dark green extract. The hexane extract was filtered and evaporated to dryness under pressure at 50°C and dissolved in DMSO for* in vitro* studies. Rotary vacuum evaporator was used to concentrate the methanol extract. The yield of the extracts was found to be 3.2% and 1.8% for the methanol and hexane extracts, respectively. The stock extracts were kept at −20°C in airtight glass container.

### 2.3. Silylation Derivatization

One milliliter of each extract was transferred to a 2 mL glass vial, and the solvents were evaporated under a stream of nitrogen at room temperature. A 150 *μ*L of* N,O*-Bis(trimethylsilyl)trifluoroacetamide (BSTFA) containing 1% trimethylchlorosilane reagent used for GC silylation derivatization (>99%, Sigma-Aldrich) was added to each dry* Gundelia tournefortii* crude extract followed by heating up to 70°C for 20 minutes [10, 22]. One microliter of each derivatized sample was injected into the gas chromatograph coupled with mass selective detector (GC/MS).

### 2.4. Gas Chromatography-Mass Spectrometry Analysis

Solutions of* Gundelia tournefortii* from methanol and hexane extracts were ran and identified using HP5890 Series II GC equipped with a Hewlett-Packard MS Engine (HP5989A) single quadrupole MS, HP7673 autosampler, HP MS-DOS Chemstation, and HP-5MS capillary column (0.25 *μ*m × 15 m × 0.25 mm) with Triple-Axis Detector. The GC was operated on an Agilent J&W GC column HP-5 column (30 m × 0.32 mm, i.d. with 0.25 *μ*m film thickness). The injection port temperature was 180°C and the initial temperature was 40°C for 6 min, followed by gradient 20°C/min until 140°C and then gradient 10°C/min until 200°C, and hold on to this temperature for 3 min. The MS parameters were 180°C for the source temperature and 280°C for the transfer line, positive ion monitoring, and EI-MS (70 eV) [10].

### 2.5. Identification of Components

The percentages of the phytochemical components were calculated from the GC peak areas by normalization. Library searches were carried out using the Mass Spectral Library of the National Institute of Standards and Technology (NIST, Gaithersburg, USA) or with mass spectra extracted from the literature [10].

### 2.6. Cell Culture

Rat L6 muscle cell lines genetically modified to express myc-tagged GLUT4 (L6-GLUT4myc) stably were maintained in myoblast monolayer culture [7]. Cells were grown under 95% air and 5% CO_2_ atmosphere in *α*-MEM accompanied with 10% fetal bovine serum (FBS), 0.1 mg/ml streptomycin, and 0.1 mU/ml penicillin.

### 2.7. MTT Assay

MTT (3-[4,5-dimethylthiazol-2-yl]-2,5-diphenyltetrazoliumbromide) is a water soluble tetrazolium salt. Once delivered to the mitochondria, it is converted to an insoluble purple formazan by succinate dehydrogenase. As such, the formazan product accumulates only in healthy cells [23]. The assay was optimized for the L6-GLUT4myc cell line as described previously [10]. Cells (2 × 10^4^/well) were plated in 200 *μ*l of medium/well in 96-well plates and were allowed to attach to the plate for 24 h. The cells were incubated then with the* Gundelia tournefortii* extracts (0-1 mg/mL) for additional 24 h. The cell medium was then replaced with 100 *μ*l fresh medium/well containing 0.5 mg/mL MTT and cultivated for 4 h darkened in the cells incubator. The supernatant was removed and 100 *μ*l isopropanol/HCl (2% HCl (0.1 M) in isopropanol) was added per well. The absorbance at 620 nm was measured with microtiter plate reader (Anthos). Two wells per plate without cells served as blank. All experiments were repeated three times in triplicate. The effect of the plants extracts on cell viability was expressed using the following formula:

(1) Percent viability=A620 nm of plant extract treated sampleA620 nm of none treated sample∗100.

### 2.8. Lactate Dehydrogenase Assay (LDH)

LDH, a cytoplasmic enzyme, release is the consequence of cell membrane breach. The activity of LDH released to the cell culture medium was monitored following the formation of formazan at 492 nm according to the manufacture kit (Promega) and was described earlier [10]. Cell membrane breach was defined as the ratio of LDH activity in the cell culture medium of treated cells relative to the LDH activity released in the control cells. L6-GLUT4myc cells (2 × 10^4^/well) were plated in 200 *μ*l of medium/well in 96-well plates and were allowed to attach to the plate for 24 h. The cells were incubated then with the* Gundelia tournefortii* extracts (0-1 mg/mL) for additional 24 h and the LDH activity in the medium was then measured. 50 *μ*l from each well was transferred to a new 96-well plate and the enzyme reaction was carried out according to the manufacture kit (CytoTox 96, Promega). The experiments were performed in triplicate. The following formula effect was used to calculate the plant extracts effect on cell viability:

(2) Percent viability=A492 nm of plant extract treated sampleA492 nm of control∗100.

### 2.9. Determination of Surface GLUT4myc

Surface myc-tagged GLUT4 was measured in intact cells as described previously [10, 24] using anti-myc antibody followed by horseradish peroxidase conjugated secondary antibody. L6-GLUT4myc cells grown in 24-well plates for 24 h followed by addition of the plant extracts for 20 h and serum-starvation for 3 h (including the plant extracts) were treated with or without 1 *μ*M insulin for 20 min. The cells were washed twice with ice-cold PBS and immediately fixed with 3% paraformaldehyde for 15 min, blocked with 3% (v/v) goat serum for 10 min, incubated with polyclonal anti-*myc *antibody (1 : 200) for 1 h at 4°C, washed 10 times with PBS and incubated with goat anti-rabbit-secondary antibody conjugated with horseradish peroxidase (1 : 1000) for 1 h at 4°C, and then washed 10 times with PBS at room temperature. One milliliter of* o*-phenylenediamine dihydrochloride reagent was added to each well and incubated in the dark at room temperature for 20–30 min. 0.5 ml of 3 M HCl was added to each well to stop the reaction. 100 *μ*l from each well was transferred to 96 well plates and the absorbance was measured at 492 nm. Background absorbance obtained from 3 wells in each 24-well plate untreated with anti-*myc *antibody was subtracted from all values.

## 3. Results and Discussion

Glucose transporter 4 (GLUT4) continuously recycles between intracellular stores (vesicles) and the plasma membrane (PM). Insulin shifts GLUT4 translocation towards the PM while glucagon shifts GLUT4 translocation towards the intracellular stores [8, 25, 26]. Several traditional used antidiabetic medicinal plants were reported to exert their hypoglycemic effects through increasing glucose transporter (GLUT) translocation to the plasma membrane in muscle, liver, and hepatic tissue [25, 26]. Although,* G. tournefortii *is recommended by herbal and integrative practitioners for the treatment of diabetes [19, 20] the action mechanism whereby* G. tournefortii *exerts its hypoglycemic effects is still unknown. Therefore, the present study was conducted to evaluate the role of GLUT4 translocation in the observed antidiabetic* G. tournefortii *effects. Two* G. tournefortii *extracts (methanol and hexane) were prepared and their effects on GLUT4 translocation were measured in L6 skeletal muscle cell line, in the present and absence of insulin. Moreover, the chemical composition of* G. tournefortii *extracts was analyzed via GC/MS, highlighting potential antidiabetic compounds in* G. tournefortii *extracts.

### 3.1. *G. tournefortii *Chemical Composition

**Figure 1:**
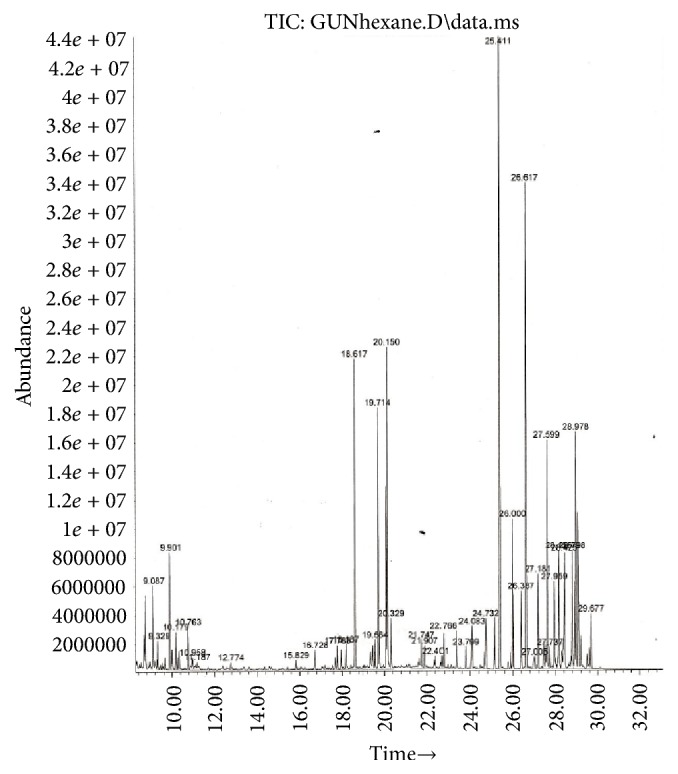
Total ion chromatogram (TIC) of* G. tournefortii *hexane extract.

**Figure 2:**
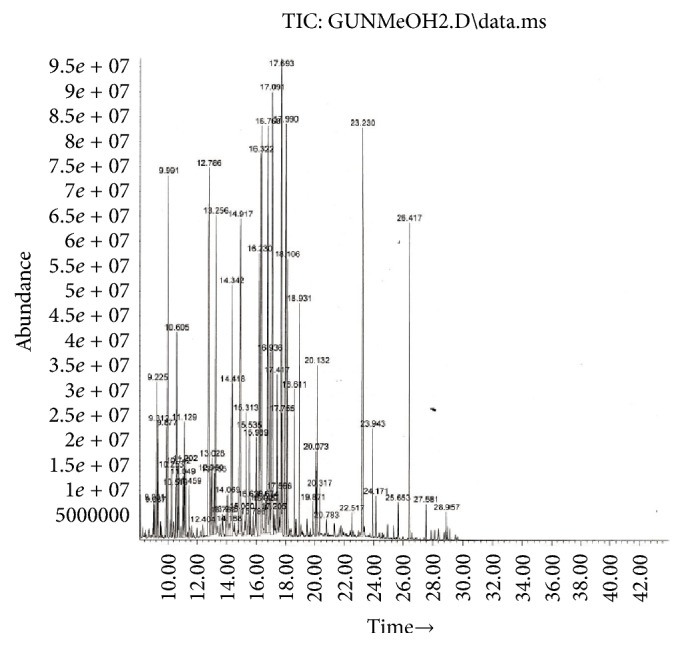
Total ion chromatogram (TIC) of* G. tournefortii *methanol extract.

Resolution, selectivity, and elution time were obtained on the capillary GC HP-5 column. We noticed sharp peaks culminated. The derivatization of the secondary metabolites of* G. tournefortii *seemed to be helpful due to presence of polar phytochemicals. Typically, upon derivatization, volatile and stable compounds are generated with amenable properties to GC/MS analysis. Figures 1 and 2 show the total ion chromatograms (TICs) of the hexane and methanol extracts.

**Figure 3:**
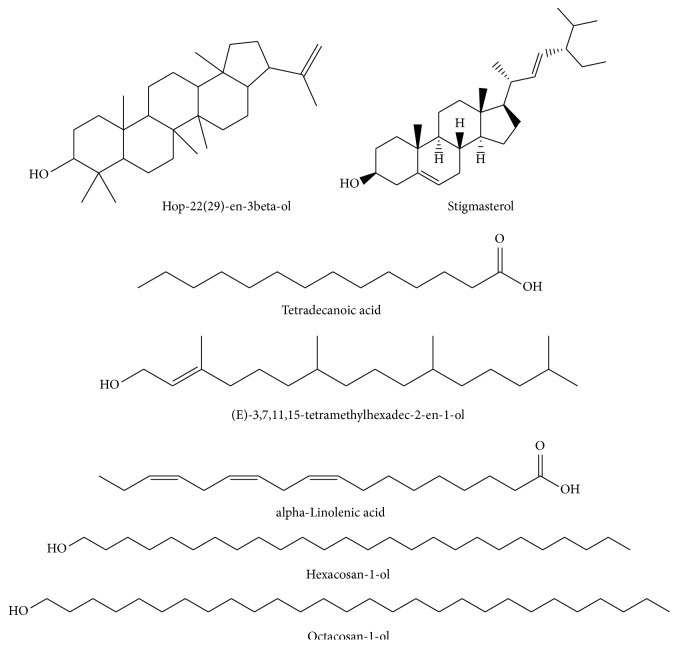
Chemical structure of major components in* G. tournefortii *hexane extract.

**Figure 4:**
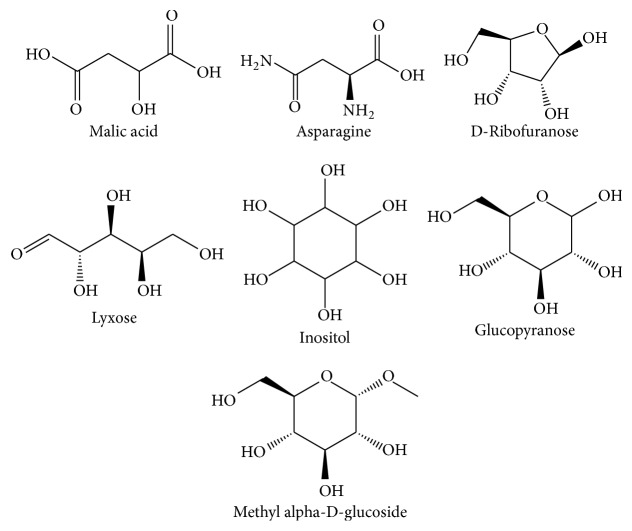
Chemical structure of major components in* G. tournefortii *methanol extract.

Phytochemical screening using GC/MS in the electron impact mode (EI) revealed 46 compounds in* G. tournefortii *methanolic (Table 1) and hexane extracts (Table 2), including sterols, esters, phenolic, saturated and unsaturated fatty acids, and aromatic compounds. 39 out of the 45 detected compounds are reported for the first time in* G. tournefortii *(Tables 1 and 2). Six components, namely, Stigmasterol (PubChem CID: 5280794), *β*-Sitosterol (PubChem CID: 222284), Palmitic acid, Linoleic acid, *α*-Linolenic acid, and Stearic acid were reported elsewhere [19]. Only one mutual compound, Stigmasterol, was mutual in the two extracts (Table 1). The GC/MS analysis revealed seven major components in each extract of the hexane and methanol (Figures 3 and 4).

### 3.2. *G. tournefortii *Extracts Toxicity

**Figure 5:**
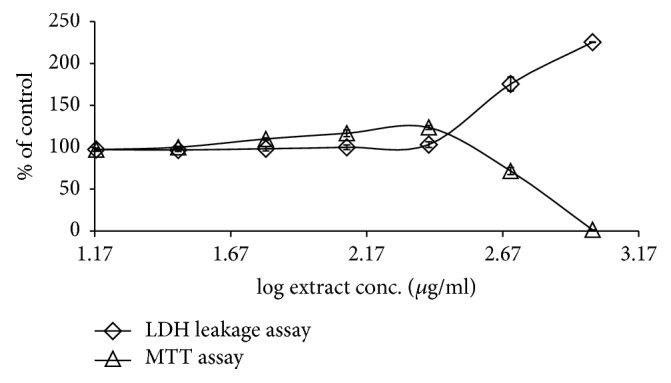
Effect of* G. tournefortii *hexane extract on cell viability by MTT and LDH leakage assays. L6-GLUT4myc cells (20,000 cell/well) exposed to methanol extract for 20 h. Values given represent means ± SEM (% of untreated control cells) of three independent experiments carried out in triplicate.

**Figure 6:**
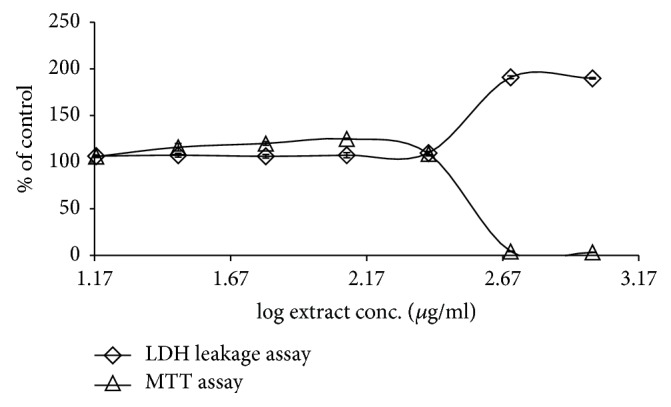
Effect of* G. tournefortii *methanol extract on cell viability by MTT and LDH leakage assays. L6-GLUT4myc cells (20,000 cell/well) exposed to hexane extract for 20 h. Values given represent means ± SEM (relative to untreated control cells) of three independent experiments carried out in triplicate.

MTT and LDH leakage assays were used to evaluate the nontoxic concentrations of the methanol and hexane* G. tournefortii *extracts. The plant extracts toxicity was tested* in vitro* in L6-GLUT4myc cells. Cells were seeded in 96 well plates and were subjected to increasing concentrations of the extracts (0-1 mg/ml) for 24 hours. Extracts concentrations that led to less than 5% cell death were considered safe. Hexane (Figure 5) and methanol (Figure 6) extracts were found to be safe up to 250 *μ*g/ml. The efficacy studies were performed at concentrations less than the safe concentration for each extract.

### 3.3. Effects of* G. tournefortii *Extracts on GLUT4 Translocation

Skeletal muscle and liver are the primary tissues responsible for dietary glucose uptake and disposal. In muscle and adipose tissues, insulin promotes the exocytic traffic of intracellular GLUT4 vessels towards the plasma membrane to elicit a rapid increase in glucose uptake [8, 25, 26]. In insulin resistance and diabetes type II, insulin fails to promote GLUT4 translocation to the PM. Some of the antidiabetic synthetic drugs and medicinal plant-based products bypass the insulin resistance by increasing GLUT4 translocation in insulin dependent or independent pathway [4].

**Figure 7:**
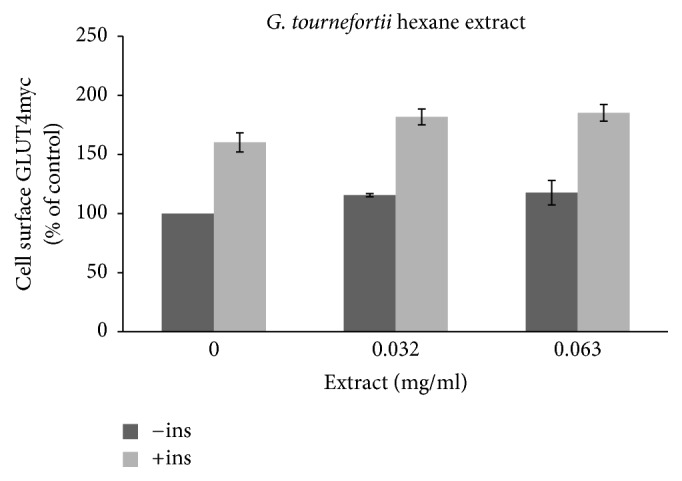
GLUT4 translocation to the plasma membrane. For the evaluation of the GLUT4 L6-GLUT4myc, cells (150,000 cell/well) were exposed to hexane extract for 20 h. Serum depleted cells were treated without (−) or with (+) 1 *μ*M insulin for 20 min at 37°C and surface* myc*-tagged GLUT4 density was quantified using the antibody coupled colorimetric assay. Values given represent means ± SEM (relative to untreated control cells) of three independent experiments carried out in triplicate.

**Figure 8:**
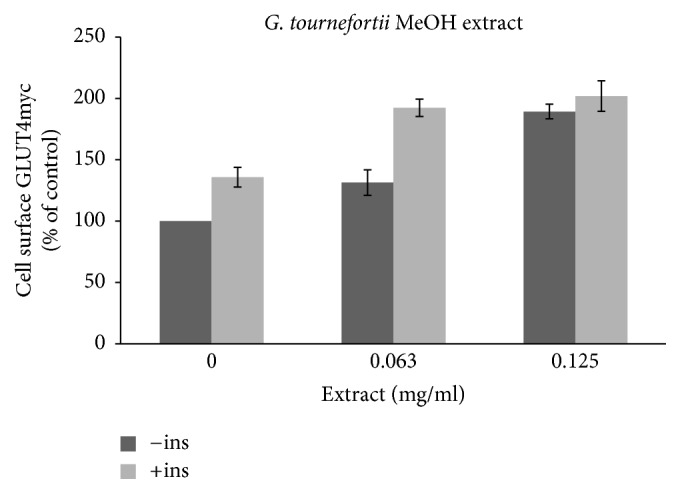
GLUT4 translocation to the plasma membrane. For the evaluation of the GLUT4 L6-GLUT4myc, cells (150,000 cell/well) were exposed to MeOH extract for 20 h. Serum depleted cells were treated without (−) or with (+) 1 *μ*M insulin for 20 min at 37°C and surface* myc*-tagged GLUT4 density was quantified using the antibody coupled colorimetric assay. Values given represent means ± SEM (relative to untreated control cells) of three independent experiments carried out in triplicate.

The involvement of glucose transporter (GLUT4) in the observed antidiabetic effects of* G. tournefortii *extracts was evaluated here by applying the GLUT4 translocation assay. Insulin increases GLUT4 translocation to the surface of myoblasts, where it mediates the increase in glucose uptake [8, 26]. L6 skeletal muscle cell lines expressing myc epitope at the exofacial loop of the glucose transporter 4 (GLUT4), named L6-GLUT4myc, were used as a model to follow GLUT4 translocation to the plasma membrane [8]. The extracts were added to the L6-GLUT4myc cells in the presence or absence of insulin and GLUT4myc translocation to the plasma membrane was assessed as described in Methods. Results obtained indicate that, in muscle L6-GLUT4myc cells, insulin-independent (basal) as well as insulin dependent GLUT4 translocation to the PM is significantly increased in response to* G. tournefortii.* extracts (especially the methanol extract). Insulin enhanced GLUT4 translocation about 150% (Figures 7 and 8) as reported elsewhere [8].

The hexane extract was found to have the lowest effects on GLUT4 translocation, and only 16% increase of GLUT4 translocation was obtained at 32 *μ*g/ml and 63 *μ*g/ml* G. tournefortii *hexane extracts in the absence of insulin. A similar effect was appreciated in the presence of insulin (Figure 7). Methanol extract (63 *μ*g/ml) increased GLUT4 translocation to the PM by about 1.5 and 2 times in the absence and presence of insulin, respectively (Figure 8).

One of the detected compounds in* G. tournefortii *was palmitic acid. Intestinally, palmitic acid was recently reported by our group to be found in three deferent* Ocimum basilicum* L. extracts (methanol, hexane and dichloromethane). Ocimum* basilicum* was reported as antidiabetic herb and palmitic acid was suggested to take an essential role in the plant extracts antidiabetic activity [10]. Concomitant with our previous reported results, palmitic acid was detected only in the MeOH extract of* G. tournefortii*, that enhanced GLUT4 translocation much more than the hexane extract.

## 4. Conclusion

The extent of increase in insulin-stimulated GLUT4 translocation was additive to that of basal GLUT4 translocation in* G. tournefortii*-exposed cells, suggesting a possible synergistic effect between* G. tournefortii *active ingredients and insulin. Alternately,* G. tournefortii *active ingredients might activate GLUT4 translocation in noninsulin dependent pathway, such as AMPK pathway. It is possible then that* G. tournefortii *active ingredients might possess “insulin-like” or “insulin-sensitizing” activity/compounds. It is essential to dissect* G. tournefortii *active compounds in order to identify its cellular molecular target and point out its specific antidiabetic mechanism and cellular pathway(s).

## Figures and Tables

**Table 1:** Phytochemicals of *Gundelia tournefortii *from methanol extract verified by GC/MS.

#	Compound name	RT (minute)	Peak area%	% of similarity^*∗*^
(1)	Aminoethanol	9.87	0.93%	91
(2)	Glycerol	9.99	3.59%	76
(3)	L-Isoleucine	10.255	0.45%	91
(4)	Succinic acid	10.6	2.2%	72
(5)	Glyceric acid	10.75	0.68%	91
(6)	Fumaric acid	11.05	0.46%	86
(7)	DL-serine	11.15	0.95%	94
(8)	2-Piperidine carboxylic acid	11.198	0.58%	94
(9)	Threonine	11.46	0.52%	90
(10)	Malic acid	12.79	7.58%	91
(11)	Asparagine	14.94	6.15%	99
(12)	Xylitol	15.05	0.28%	83
(13)	Arabitol	15.32	1.12%	94
(14)	D-Ribofuranose	16.23	3.96%	74
(15)	D-Galactofuranose	16.625	0.45%	80
(16)	Lyxose	17.1	6.88%	80
(17)	Sorbitol	17.42	1.36%	91
(18)	Inositol	17.7	5.09%	93
(19)	Glucopyranose	18.00	5.01%	89
(20)	D-Gluconic acid	18.15	2.61%	70
(21)	Palmitic acid	18.61	0.95%	70
(22)	Linoleic acid	20.069	0.66%	91
(23)	L-Tryptophan	20.135	1.22%	87
(24)	Methyl *α*-D-glucopyranoside	23.23	7.04%	72
(25)	D-Xylonic acid	24.17	0.33%	83
(26)	*Stigmasterol*	27.58	0.24%	99

^*∗*^% of similarity relative to the reference library in the GC/MS.

**Table 2:** Phytochemicals of *Gundelia tournefortii *from hexane extract verified by GC/MS.

Number	Component name	RT (minutes)	Peak area%	% of similarity^*∗*^
(1)	Propanoic acid	12.77	0.34%	64
(2)	Cetanol	17.78	1.48%	94
(3)	Ethyl icosanoate	18.18	0.58%	87
(4)	Tetradecanoic acid	18.613	5.06%	64
(5)	Octadecan-1-ol	19.556	2.2%	97
(6)	(E)-3,7,11,15-Tetramethylhexadec-2-en-1-ol	19.714	4.68%	86
(7)	*α*-Linolenic acid	20.152	9.38%	99
(8)	Stearic acid	20.317	0.95%	80
(9)	Oleamide	21.74	1.37%	94
(10)	Eicosanoic acid	21.906	0.71%	72
(11)	5-Octadecene	22.402	0.51%	87
(12)	Di-n-Octyl Phtalate	22.76	1.99%	72
(13)	Tetracosan-1-ol	24.09	1.09%	90
(14)	Hexacosan-1-ol	25.414	15.07%	81
(15)	Heptacosane	26.39	1.05%	91
(16)	Octacosan-1-ol	26.623	9.15%	97
(17)	*Stigmasterol*	27.599	4.73%	96
(18)	*β*-sitosterol	27.95	1.91%	66
(19)	12-Oleanen-3-yl acetate	28.791	2.69%	94
(20)	Hop-22(29)-en-3beta-ol	29.67	1.97%	80

^*∗*^% of similarity relative to the reference library in the GC/MS.
